# Supplementary material for: Health extension workers’ perceived health system context and health post preparedness to provide services: a cross-sectional study in four Ethiopian regions
Source: BMJ Open. 2021 Jun 9;11(6):e048517. doi: 10.1136/bmjopen-2020-048517 (PMC8191611; doi:10.1136/bmjopen-2020-048517)
Supplement: Supplementary data [file bmjopen-2020-048517supp001.pdf]

Supplemental Table S1. Association between context dimensions and interventions and comparison areas in four regions of Ethiopia, 2018 (N=152).

Dimensions		Comparison (N=74)	Intervention (N=78)	Fisher's exact test
Resource	Agree	2.7	2.6	1
	Disagree	97.3	97.4	
Community engagement	Agree	78.4	88.5	0.125
	Disagree	21.6	11.5	
Monitoring service for action	Agree	58.1	73.1	0.061
	Disagree	41.9	26.9	
Source of knowledge	Agree	2.7	2.7	1
	Disagree	97.3	97.3	
Commitment to work	Agree	66.2	75.6	
	Disagree	33.8	24.4	0.215
Work culture	Agree	64.9	66.7	
	Disagree	35.1	33.3	0.865
Leadership	Agree	60.8	66.7	
	Disagree	39.2	33.3	0.501
Informal payment	Agree	1.4	1.3	1
	Disagree	98.7	98.7	
