# Supplementary material for: Health extension workers’ perceived health system context and health post preparedness to provide services: a cross-sectional study in four Ethiopian regions
Source: BMJ Open. 2021 Jun 9;11(6):e048517. doi: 10.1136/bmjopen-2020-048517 (PMC8191611; doi:10.1136/bmjopen-2020-048517)
Supplement: Author's manuscript [file bmjopen-2020-048517.draft_revisions.pdf]

BMJ Open is committed to open peer review. As part of this commitment we make the peer review history of every article we publish publicly available.

When an article is published we post the peer reviewers' comments and the authors' responses online. We also post the versions of the paper that were used during peer review. These are the versions that the peer review comments apply to.

The versions of the paper that follow are the versions that were submitted during the peer review process. They are not the versions of record or the final published versions. They should not be cited or distributed as the published version of this manuscript.

BMJ Open is an open access journal and the full, final, typeset and author-corrected version of record of the manuscript is available on our site with no access controls, subscription charges or pay-per-view fees (http://bmjopen.bmj.com).

If you have any questions on BMJ Open's open peer review process please email info.bmjopen@bmj.com

BMJ Open

Health extension workers' perceived health system context and health post preparedness to provide services: A cross-sectional study in four Ethiopian regions

Journal:	*BMJ Open*
Manuscript ID	bmjopen-2020-048517
Article Type:	Original research
Date Submitted by the Author:	30-Dec-2020

Complete List of Authors:	Getachew, Theodros ; Ethiopian Public Health Institute; University of Gondar, Institute of Public Health, College of Medicine and Health Science<br>Abebe, Solomon; University of Gondar, Institute of Public Health, College of Medicine and Health Science<br>Yitayal, Mezgebu ; University of Gondar, Health Services Management and Health Economics<br>Bergström, Anna; Uppsala Universitet<br>Persson, Lars; London School of Hygiene and Tropical Medicine; Ethiopian Public Health Institute<br>Berhanu, Della; London School of Hygiene & Tropical Medicine, Disease Control; Ethiopian Public Health Institute

Keywords:	Quality in health care < HEALTH SERVICES ADMINISTRATION & MANAGEMENT, Organisation of health services < HEALTH SERVICES ADMINISTRATION & MANAGEMENT, International health services < HEALTH SERVICES ADMINISTRATION & MANAGEMENT

SCHOLARONE™
Manuscripts

I, the Submitting Author has the right to grant and does grant on behalf of all authors of the Work (as defined in the below author licence), an exclusive licence and/or a non-exclusive licence for contributions from authors who are: i) UK Crown employees; ii) where BMJ has agreed a CC-BY licence shall apply, and/or iii) in accordance with the terms applicable for US Federal Government officers or employees acting as part of their official duties; on a worldwide, perpetual, irrevocable, royalty-free basis to BMJ Publishing Group Ltd ("BMJ") its licensees and where the relevant Journal is co-owned by BMJ to the co-owners of the Journal, to publish the Work in this journal and any other BMJ products and to exploit all rights, as set out in our licence.

The Submitting Author accepts and understands that any supply made under these terms is made by BMJ to the Submitting Author unless you are acting as an employee on behalf of your employer or a postgraduate student of an affiliated institution which is paying any applicable article publishing charge ("APC") for Open Access articles. Where the Submitting Author wishes to make the Work available on an Open Access basis (and intends to pay the relevant APC), the terms of reuse of such Open Access shall be governed by a Creative Commons licence – details of these licences and which Creative Commons licence will apply to this Work are set out in our licence referred to above.

Other than as permitted in any relevant BMJ Author's Self Archiving Policies, I confirm this Work has not been accepted for publication elsewhere, is not being considered for publication elsewhere and does not duplicate material already published. I confirm all authors consent to publication of this Work and authorise the granting of this licence.

For peer review only

Health extension workers' perceived health system context and health post preparedness to provide services: A cross-sectional study in four Ethiopian regions

Theodros Getachew[1,2], Solomon Mekonnen Abebe[2], Mezgebu Yitayal[2], Anna Bergström[3], Lars Åke Persson[1, 4], Della Berhanu[1, 4]

1. Health System and Reproductive Health Research Directorate, Ethiopian Public Health Institute, Addis Ababa, Ethiopia
2. College of Medicine and Health Sciences, Institute of Public Health, University of Gondar, Gondar, Ethiopia
3. Uppsala Global Health Research on Implementation and Sustainability (UGHRIS), Department of Women and Children Health, Uppsala University, Uppsala, Sweden
4. Department of Disease Control, London School of Hygiene & Tropical Medicine, Keppel Street, London, United Kingdom

Correspondence to:

Email: tedi.getachew@yahoo.com

For peer review only

Word count: 2,762

For peer review only - http://bmjopen.bmj.com/site/about/guidelines.xhtml

ABSTRACT

Context and objective The health system context influences the implementation of evidence-based practices and quality of health care services. Ethiopia aims at reaching universal health coverage but faces low primary care utilization and substandard quality-of-care. We assessed the health extension workers' perceived context and the preparedness of health posts to provide services.

Design Cross-sectional study

Setting This study was part of evaluating a complex intervention in 52 districts of four regions of Ethiopia. Data were collected from December 2018 to February 2019. A total of 152 health posts serving selected enumeration areas in these districts were included.

Outcome measures We used the Context Assessment for Community Health (COACH) tool and the Service Availability and Readiness Assessment (SARA) tool.

Results Internal reliability of COACH was satisfactory. The dimensions *community engagement*, *work culture*, *commitment to work*, and *leadership* all scored high (mean 3.75-4.01 on a 1-5 scale), while *organizational resources, sources of knowledge,* and *informal payments* scored low (1.78-2.71). The general service readiness index was 59%. On average, 67% of the health posts had basic amenities to provide services, 81% had basic equipment, 42% had standard precautions for infection prevention, 47% had test capacity for malaria, and 58% had essential medicines.

Conclusion The health extension workers had a good relationship with the local community, used data for planning, were highly committed to their work with positive perceptions of their work culture, a relatively positive attitude regarding their leaders, and reported no corruption or informal payments. In contrast, they had insufficient sources of information and a severe lack of resources. The health post preparedness confirmed the low level of resources and preparedness for services. These findings suggest a significant potential contribution by health extension workers to Ethiopia's primary health care, provided that they receive improved support, including new information and essential resources.

Key words: Health system context, facility preparedness, health extension workers

For peer review only

Strengths and limitations of this study

- This study was the first assessment of Ethiopian front-line primary healthcare workers' perceived health system context and the facility preparedness for services.
- Understanding the health system context increases the likelihood of successful implementation of evidence-based practices.
- The Context Assessment for Community Health (COACH) tool has been validated in a range of other low-income countries and was found to have satisfactory internal reliability when translated into three Ethiopian languages.
- Although precautions were taken to obtain valid responses from the interviewed health extension workers, we cannot exclude the risk of respondents' bias.
- The sample represented 52 districts in four Ethiopian regions that participated in a child health services study, and inferences cannot be drawn to the whole country.

For peer review only - http://bmjopen.bmj.com/site/about/guidelines.xhtml

INTRODUCTION

The health system context is essential for new interventions and quality-of-care (1). Health care of poor quality contributes to the high mortality in low- and middle-income countries (2,3). These quality problems have multiple causes, for example, lack of resources and suboptimal interaction between health care providers and clients (4). Thus, there is a growing understanding that the health system context matters for efforts to improve health services quality (5,6). However, we lack consensus on the definition, operationalization, and methods to study context (1).

Therefore, we need systematic ways of assessing the context in which health workers are active (7). The most frequently used tools and information systems provide structural information, for example, the Service Availability and Readiness Assessment (SARA) tool (8). Mapping the facility preparedness sets the scene, but such assessments are poorly associated with the quality of services provided (9). A meta-review showed that access to information, community engagement, leadership, regulations and standards, organizational capacity, models of care, communication, and work satisfaction are essential for implementing new interventions and quality-of-care (6,10). The Context Assessment for Community Health (COACH) tool was developed and validated in low- and middle-income countries and included many of the context dimensions mentioned above (11).

In Ethiopia, under-five children's primary level services are provided through the health extension program (12). Two female community health workers, known as health extension workers, provide preventative and curative services for a population of 5000. They offer static services from health posts as well as outreach services within the community. Five health posts, their referral health centre, and a primary district-level hospital comprise the primary health care unit (13). Despite the successful implementation of the health extension program, the program is currently facing challenges that

For peer review only

For peer review only - http://bmjopen.bmj.com/site/about/guidelines.xhtml

remain to be addressed. These challenges are related to the utilization and quality of services offered by the health extension workers and their working and living conditions (14).

The Ethiopian Ministry of Health aims to increase the primary health care services' access and quality through reforms and new initiatives (15,16). One such effort was the Optimizing the Health Extension Program intervention to increase the quality and utilization of under-five health services. As part of the evaluation of that intervention, we have shown that health extension workers did not follow the clinical guidelines for assessing and managing sick children with common illnesses (17). Their ability to classify childhood illnesses was also low (18). Unfortunately, the intervention, which included community engagement, training, supportive supervision, and performance reviews of health workers, neither increased care-seeking for sick children (19) nor improved the classification of childhood illnesses by these primary health care workers (Getachew T et al, submitted manuscript). The failure of such an intervention could, at least partly, be attributed to the context of the health extension workers. There is a need for accurate measurements that reflect the health system context in which care is provided to patients and populations (20). Therefore, we aimed to assess the health extension workers' perceived health system context and the health posts' service readiness in four Ethiopian regions.

METHODS

Study setting and design

This study was part of a large project, which assessed a complex intervention's effectiveness to increase care-seeking for children under five years. This intervention had three components: community engagement, capacity building, and ownership and accountability of child health services. The assessment was done in 52 districts of four regions (Amhara, Tigray, Oromia, Southern Nations, Nationalities, and Peoples) of Ethiopia with baseline and end-line surveys conducted before and after

For peer review only - http://bmjopen.bmj.com/site/about/guidelines.xhtml

the intervention. This paper used the endline data that was conducted from December 2018 to February 2019. The protocol and results of the evaluation have been published (19,21).

Subjects

A total of 200 enumeration areas were selected to represent the selected districts in the end-line survey. Health posts serving these areas were included in the study, and their preparedness for services was assessed. One health extension worker at each health post was interviewed, and their perceived context was evaluated.

Study tools

Two tools were used at facility and provider levels. The provider-level tool aimed to assess the health extension workers' perceived context on the service delivery environment. This tool, labelled the Context Assessment for Community Health (COACH), has 49 items that measure eight dimensions of context (Table 1) (22). The tool was developed in Bangladesh, Vietnam, Uganda, South Africa, and Nicaragua (11). It also includes demographic questions on age, gender, professional qualification, health facility, and years working at the current facility. The tool items were measured on a five-point Likert scale ranging from 'strongly disagree' to 'strongly agree'. Items in *source of information* were measured for availability and frequency of use. The Brislin model (23) was used to translate the tool into Amharic, Oromiffaa, and Tigrigna, including forward translation, review of the translated tool, backward translation, and comparison of the original and back-translated tools. Conrad and Blair's taxonomy (24) was used to describe the problems that appeared in the translations. Accordingly, there were six lexical problems with difficulties in the meanings of words, one logical problem, and one inclusion or exclusion problem. All identified translation problems were possible to correct.

Table 1. Definitions of context assessment dimensions in the Context Assessment for Community Health tool.

For peer review only - http://bmjopen.bmj.com/site/about/guidelines.xhtml

Dimensions	Number of items	Definition
Resources	11	The availability of resources (staff, space, time, communication and transport, drugs, equipment and supplies, finance) that allows a unit to adapt successfully to internal and external pressures.
Community engagement	5	The mutual communication, deliberation and activities that occur between community members and units.
Monitoring services for action	5	The process of using data to assess group/team performance.
Sources of knowledge	5	The structures that facilitate the ability to access and use knowledge.
Commitment to work	3	The relative strength of an individual's identification with and involvement in a particular work organization.
Work culture	6	The way that 'we do things' in our organizations and work units. This includes culture of learning and change, and culture of responsibility.
Leadership	6	The actions of formal leaders in an organization (unit).
Informal payment	8	Payments to individuals, which are made outside official payment channels including nepotism, and accountability.

The facility tool aimed at collecting information on the overall facility-level preparedness to provide child health services (Table 2), which was based on the World Health Organization Service Availability and Readiness Assessment reference manual (25). The tool was translated into three local languages (Amharic, Oromiffaa and Tigrigna), pretested and amended.

Table 2. Domains and their items used to construct the health post service availability and readiness.

Domain	Items
Basic amenities	Communication equipment
	Access to adequate sanitation facilities for clients
	Improved water source
	Power supply
Basic equipment	Infant scale
	Thermometer
	Functional stethoscope
	Mid-upper arm circumference tape measure
Standard precautions	Sharps container
	Chlorine bleach
	Bucket for decontamination solution
	Contaminated waste container

For peer review only

For peer review only - http://bmjopen.bmj.com/site/about/guidelines.xhtml

	Soap and towel or hand rub
	Alcohol-based hand rub
	Clean glove
Diagnostics	Malaria rapid diagnostic test
Essential medicines	Vitamin A
	Gentamycin
	Amoxicillin susp/tab
	Oral rehydration solution
	Zinc
	Coartem (Artemether Lumefantrine)
	Ready-to-use therapeutic food

For peer review only

Measurements

We judged the COACH tool's internal consistency with Cronbach alpha (26) that expressed if items in the instrument's different dimensions measured the same thing. Descriptive statistics were used to assess the health extension workers' agreement to the items and dimensions. All items except for source of knowledge were measured on a scale of 1-5, where the scores for item 48 and 49 were reversed to measure in the same direction as other items. The overall agreement was a calculated value drawn by multiplying the number of items in the dimension by four, which was coded as agreement. An individual was considered to agree if her score was above the calculated value.

The general health service readiness score was a composite summary measure calculated by combining information from the five general service readiness domains: basic amenities, standard precautions for infection prevention, basic equipment, diagnostics, and essential medicines (25). For each domain, the average availability of tracer items was revealed as the domain score. Each dimension's mean score was computed to assess the average responses to the included items in the dimension. The analysis was performed using STATA 14.2 statistical package (Stata Corp LP, College Station, TX, USA).

RESULTS

For peer review only - http://bmjopen.bmj.com/site/about/guidelines.xhtml

A total of 165 health posts were assessed, and 154 health extension workers were available for interview. After merging the two datasets, 152 health post and health extension worker data were available for analysis.

Perceived context

Table 3 presents the average interitem correlation and the Cronbach Alpha coefficients for the eight context dimensions. Almost all dimensions exceeded the commonly accepted standard for satisfactory internal reliability (0.70) for new scales (α range = 0.51 to 0.89). One dimension (*source of knowledge*, α = 0.51) did not meet this standard. The average inter-item correlation ranged from 0.17 to 0.59. The ideal range of average inter-item correlation is 0.15 to 0.50; less than 0.15 indicates that items are not well correlated and don't measure the same idea very well. More than 0.50 means that items are close, almost repetitive.

The mean scores of the COACH dimensions on a scale of 1-5 are presented in table 3. The dimensions *community engagement*, *work culture*, *commitment to work*, and *leadership* all scored high (mean 3.75-4.01 on the 1-5 scale), while *organizational resources, sources of knowledge,* and *informal payments* scored low (1.78-2.71). These findings indicate that the health extension workers neither perceived themselves as having sufficient resources to conduct their work nor to have access to new knowledge.

Table 3. Summary of perceived context of health extension workers and the internal consistency of the Context Assessment for Community Health tool. Survey in four Ethiopian regions, 2018. (N=152)

Dimensions	Number of items	Mean (SD)	Cronbach alpha	Average interitem correlation
Resources	11	2.60 (0.60)	0.7620	0.2255
Community engagement	5	4.01 (0.58)	0.8813	0.5975
Monitoring services for action	5	3.75 (0.70)	0.8678	0.5676
Sources of knowledge	5	2.71 (0.79)	0.5053	0.1696
Commitment to work	3	3.79 (0.79)	0.7976	0.5677
Work culture	6	3.89 (0.51)	0.7683	0.3559
Leadership	6	3.79 (0.60)	0.8771	0.5432

For peer review only - http://bmjopen.bmj.com/site/about/guidelines.xhtml

Informal payment	8	1.78 (0.56)	0.8427	0.4011

Table 4 depicts the percentage of each item included in the eight context dimensions. Most of the health extension workers reported disagreement on the availability of financial resources. They also disagreed to having access to communication and transport.

Table 4. Percentage of items and dimensions of the Context Assessment for Community Health tool in four Ethiopian regions, 2018 (N=152).

Resource	Disagree	Neutral	Agree
1. My unit has enough workers with the right training and skills to do everything that needs to be done.	52	3	45
2. My unit has enough workers with the right training and skills to do their job in the best possible way.	52	2	46
3. My unit has enough space to provide healthcare services.	51	2	47
4. My unit has access to the transport and fuel that are needed to provide healthcare services.	88	0	13
5. My unit has access to the communication tools (e.g., telephone or radio) that are needed to provide healthcare services.	84	2	14
6. My unit has enough medicine to provide healthcare services.	48	2	50
7. My unit has enough functional equipment, such as a thermometer and blood pressure cuff, to provide healthcare services.	49	4	47
8. My unit has enough disposable medical equipment, such as syringes, gloves and needles, to provide healthcare services.	30	0	70
9. If the workload increases, my unit can get additional resources such as medicine and equipment.	45	2	53
10. My unit receives money according to an established financial plan.	84	2	14
11. My unit has money that we can decide how to use.	91	3	6
Community engagement			
12. In my unit we ask community members what they think about the healthcare services that we provide.	7	0	93
13. In my unit we listen to what community members think about the healthcare services we provide.	4	1	95
14. In my unit we have meetings with community members to discuss health matters.	5	1	93
15. In my unit we encourage community members to contribute to improving the health of the community.	3	1	96
16. In my unit we encourage other organizations to contribute to improving the health of the community.	11	0	89
Monitoring services for action			
17. I receive regular updates about my unit's performance based on information/data collected from our unit.	14	3	84
18. My unit discusses information/data from our unit in a regular, formal way, such as in regularly scheduled meetings.	11	7	82
19. My unit regularly uses unit information/data to make plans for improving its healthcare services.	13	4	84
20. My unit regularly monitors its work by comparing it with the unit's action plans.	13	5	83
21. My unit regularly compares its work with national or other guidelines.	16	2	82
Commitment to work			
27. I am proud to work in this unit.	21	3	76
28. I am satisfied to work in this unit.	16	5	80
29. I feel encouraged to do my very best at work.	7	3	89
Work culture			

For peer review only - http://bmjopen.bmj.com/site/about/guidelines.xhtml

30. My unit is willing to use new healthcare practices such as guidelines and recommendations.	4	1	95
31. My unit helps me to improve and develop my skills.	28	2	70
32. I am encouraged to seek new information on healthcare practices.	20	3	78
33. My unit works for the good of the clients and puts their needs first.	6	1	93
34. Members of the unit feel personally responsible for improving healthcare services.	6	0	94
35. Members of the unit approach clients with respect.	2	2	96
Leadership			
36. I trust the unit leader.	7	3	91
37. The leader handles stressful situations calmly.	12	4	84
38. The leader actively listens, acknowledges, and then responds to requests and concerns.	11	4	85
39. The leader effectively resolves any conflicts that arise.	14	5	82
40. The leader encourages the introduction of new ideas and practices.	13	4	83
41. The leader makes things happen.	11	5	85
Informal payment			
42. Clients must always give informal payment to health workers to access healthcare services.	97	1	3
43. Clients are treated more quickly if they make informal payments to health workers.	98	0	2
44. Medicines or equipment that should be available for free to clients have been sold in my unit.	97	1	3
45. Health workers are sometimes absent from work earning money at other places.	97	1	2
46. Health workers in my unit give healthcare services to friends and family first.	95	1	3
47. Health workers in my unit give jobs or other benefits to friends and family first.	97	1	3
48. Efforts are made to stop clients from providing informal payment to get appropriate healthcare services.	21	7	72
49. Efforts are made to stop health workers from asking clients for informal payment.	21	8	71

Sources of knowledge	Not available	Never, 0 times	Rarely 1-5 times	Occasionally 6-10 times	Frequently 11-15 times	Almost always 16 times or more
22. Clinical practice guidelines	15	4	18	16	16	32
23. Other printed material for work (e.g., textbooks, journals)	18	9	25	30	14	5
24. The Internet	68	25	5	1	0	1
25. Electronic decision support (e.g., mobile phone applications or other electronic devices to assist with care and decision-making)	56	30	3	5	5	2
26. In-service training/ workshops/courses	25	14	19	28	10	4

Figure 1 depicts the percentage of average scores for the context dimensions. Very few (2.6%) perceived their facility to have enough resources available to manage their work. Most respondents (83.6%) perceived that their facility had active communication with members of their communities. Sixty-six percent on average responded agreement for the *work culture* dimension, implying that they considered their *work culture* to support learning, change, and responsibility. A very high proportion

For peer review only - http://bmjopen.bmj.com/site/about/guidelines.xhtml

of respondents (98.7%) regarded *informal payment* for health workers not to be acceptable in their facility.

Figure 1: Percent agreement to dimensions in the Context Assessment for Community Health tool. Health extension workers in four Ethiopian regions, 2018 (N=152).

There was no difference in context dimensions between intervention and comparison areas in the evaluation's end-line survey (all $p > 0.05$). The exact percentage for each item is found Supplemented (table S1).

General facility-level readiness

Figure 2 shows the general service readiness index and domain scores. The general service readiness index was 59%, implying that six in ten health posts were ready to provide child health services. On average, about two-thirds (67%) of health posts had basic amenities to provide services, 81% had basic equipment required, 42% had standard precautions for infection prevention, 47% had diagnostic test capacity for malaria RDT, and 58% had essential medicines. The basic equipment mean score was the highest across the five domains, and the diagnostic mean score index was the lowest.

Figure 2 also shows the percentage of health posts having all tracer items available to provide general child health services. Accordingly, only 1% of health posts had all essential medicines. Half of the health posts had all tracer items for basic equipment. Three in ten health posts had all items for basic amenities.

Figure 2: Percentage of health posts with tracer items available to provide general service in four regions of Ethiopia, 2018 (N=152)

DISCUSSION

For peer review only - http://bmjopen.bmj.com/site/about/guidelines.xhtml

For peer review only

We have described the Ethiopian health extension workers' perceived context and the health posts' preparedness to provide child health services. The health extension workers perceived that they had a good relationship with the local community. They were active in using data for planning and performance, were highly committed to their work and had positive perceptions of their work culture. They also had a relatively positive experience of their leaders and reported no corruption or informal payments. In contrast, they reported having insufficient information sources and a severe lack of resources to perform their work. The latter was also reflected in the health post preparedness assessment, which overall was on a low level.

So far, there is no consensus on defining or assessing the health system context (27). Several contextual factors are associated with quality improvement, like leadership, organizational culture, information system, and organizational structure. However, there are uncertainties regarding definitions and measurements (28). Qualitative studies have contributed to the understanding of the health system context and quality of care. In this study, we quantified the perceived context and compared it with health post preparedness. The COACH tool was developed in five countries. Later, it has been used in Mozambique (29), and now in four different Ethiopian regions and three languages. Except for the sources of knowledge dimension, all other COACH tool dimensions showed good internal reliability.

The health extension workers lacked sources of new knowledge. Internet, e-health, or m-health applications were absent (29). Importantly, their responses indicated that they lacked in-service training, workshops, and courses. Insufficient sources of knowledge could lead to inappropriate diagnosis and mismanagement, such as the irrational use of antibiotics. An earlier study conducted in the same study area indicated that the health extension workers' clinical assessment, classification, and management of sick children did not follow the clinical guidelines (17). This low adherence could

For peer review only - http://bmjopen.bmj.com/site/about/guidelines.xhtml

lead to misdiagnoses and a lack of potentially life-saving treatments. Capacity building could be achieved through refresher training, followed by supportive supervision.

The health extension workers reportedly had good contact with the community they served. This engagement could help to enhance the health extension workers' accountability and dedication. A study conducted in southern Ethiopia indicated that with focused training, guidance, and regular supportive supervision, the health extension workers enhanced community participation (30). A qualitative study in southern Ethiopia revealed that health extension workers' relationships with the community could be constrained due to inadequate support systems, lack of trust, communication, dialogue, and differing expectations (31).

We also found that commitment to work was relatively good. A combination of financial and non-financial incentives is required to support motivation and satisfaction (32). Non-financial incentives, such as creating career opportunities, may increase the motivation and retention of health extension workers (33).

Informal payments were perceived to be very rare. Informal payments or various forms of corruption could have significant adverse effects on the health system, affecting patients and service providers (34). A study in Tanzania showed that informal payments existed and were negatively associated with job satisfaction and motivation (35).

The health posts in the study area seemed to have moderate service readiness, especially basic amenities and equipment. However, health posts did not have the essential medicines to provide child care. These facilities are the first contact for primary care, which provides basic health and medical care close to the community, especially in rural populations (14). These first-line services can potentially respond to a range of health challenges in low-income countries (36), but only if proper

For peer review only

For peer review only - http://bmjopen.bmj.com/site/about/guidelines.xhtml

attention is given to needs, such as essential medicines, in addition to infrastructure and basic equipment. To meet such requirements is vital for a resilient health system (37).

We have earlier shown that health extension workers' ability to classify childhood illnesses was low (18). The evaluation of the Optimizing the Health Extension Program intervention's effectiveness showed no effect on the utilization of services for sick children (19). The lack of effect could partly be attributed to delays, interruptions, and an overall short implementation period of a complex intervention. Complex interventions that aim to change health services and care-seeking for sick children may need an extended implementation period (38). Lack of effect could also be due to some of these contextual factors necessary for improving quality.

The context in which the services are provided is essential for implementing changes or new programmes. However, contextual factors are generally not well understood. Before this study, no assessments of different aspects of the health system context have been done in Ethiopia. A study conducted in six European countries found that structure and process indicators explained more variability in client satisfaction than contextual factors (39). A systematic review concluded that contextual factors might influence the effectiveness of quality improvement interventions (40).

We report the first study in Ethiopia of primary healthcare workers' perceived health system context. The Context Assessment for Community Health tool has been validated in a range of other low-income countries (11) and was also found to have satisfactory internal reliability when translated into three Ethiopian languages. Understanding context can identify factors that promote or hinder the implementation of evidence-based practices, increasing the likelihood of successful implementation. Although precautions were taken to obtain valid responses from the interviewed health extension workers, the results could be susceptible to bias. The sample represented a large number of districts

For peer review only - http://bmjopen.bmj.com/site/about/guidelines.xhtml

in four Ethiopian regions that participated in a child health services study, but inferences cannot be drawn to the whole country.

CONCLUSION

The Ethiopian health extension workers' perceived context showed a severe lack of resources. They perceived a good relationship with the local community, used data for planning but lacked access to new knowledge. They were highly committed to work and had positive perceptions of their work culture and a relatively positive attitude regarding their leaders. There was no corruption or informal payments at their work sites. The internal consistency of the context assessment tool provided evidence of its ability to measure its different dimensions. This feature will allow for tailoring implementation strategies and assessing context as part of evaluations. The health extension workers' perceptions of sources of information and available resources were in line with the results of the health facility preparedness.

For peer review only

REFERENCES

1. Rogers L, De Brún A, McAuliffe E. Defining and assessing context in healthcare implementation studies: A systematic review. BMC Health Serv Res. 2020;20(1):1–24.
2. Kruk ME, Gage AD, Joseph NT, Danaei G, García-Saisó S, Salomon JA. Mortality due to low-quality health systems in the universal health coverage era: a systematic analysis of amenable deaths in 137 countries. Lancet. 2018 Nov 17;392(10160):2203–12.
3. Chou VB, Walker N, Kanyangarara M. Estimating the global impact of poor quality of care on maternal and neonatal outcomes in 81 low- And middle-income countries: A modeling study. PLoS Med. 2019;16(12).
4. Mosadeghrad AM. Factors affecting medical service quality. Iran J Public Health. 2014;43(2):210–20.
5. Mcdonald, Kathryn M, Ferguson M. Considering Context in Quality Improvement Interventions and Implementation : Concepts , Frameworks , and Application. Acadamic Pediatr. 2013;13(6):S45–53.
6. Akachi Y, Kruk ME. Quality of care : measuring a neglected driver of improved health. Bull World Health Organ. 2017;95:465–72.
7. Daivadanam M, Ingram M, Annerstedt KS, Parker G, Bobrow K, Dolovich L, et al. The role of context in implementation research for non-communicable diseases: Answering the "how-to" dilemma. PLoS One. 2019;14(4):1–22.
8. World Health Organization. Service Availability and Readiness Assessment (SARA): An annual monitoring system for service delivery Reference Manual. In Geneva: World Health Organization; 2013.
9. Leslie HH, Sun Z, Kruk ME. Association between infrastructure and observed quality of care in 4 healthcare services : A cross-sectional study of 4 , 300 facilities in 8 countries. PLOS Med.

For peer review only - http://bmjopen.bmj.com/site/about/guidelines.xhtml

2017;14(12):e1002464.

10. Nair M, Yoshida S, Lambrechts T, Boschi-Pinto C, Bose K, Mason EM, et al. Facilitators and barriers to quality of care in maternal, newborn and child health: A global situational analysis through metareview. BMJ Open. 2014;4(5).

11. Bergström A, Skeen S, Duc DM, Blandon EZ, Estabrooks C, Gustavsson P, et al. Health system context and implementation of evidence-based practices—development and validation of the Context Assessment for Community Health (COACH) tool for low- and middle-income settings. Implement Sci. 2015;10(1).

12. Gebrehiwot TG, Sebastian MS, Edin K, Goicolea I. The Health Extension Program and its association with change in utilization of selected maternal health services in tigray region, Ethiopia: A segmented linear regression analysis. PLoS One. 2015;10(7):1–15.

13. Yitayal M, Berhane Y, Worku A, Kebede Y. Health extension program factors , frequency of household visits and being model households , improved utilization of basic health services in Ethiopia. BMC Health Serv Research. 2014;14(1):1–9.

14. Assefa Y, Gelaw YA, Hill PS, Taye BW, Van Damme W. Community health extension program of Ethiopia, 2003-2018: Successes and challenges toward universal coverage for primary healthcare services. Global Health. 2019;15(1):1–11.

15. Argaw MD, Desta BF, Bele TA, Ayne AD. Improved performance of district health systems through implementing health center clinical and administrative standards in the Amhara region of Ethiopia. BMC Health Serv Res. 2019;19(1):1–13.

16. Ministry of Health. Ethiopian Health Sector Transformation Plan.2015/16 - 2019/20. Vol. 20, Federal Democratic Republic of Ethiopia Ministry of Health. 2015.

17. Daka DW, Wordofa MA, Woldie M, Persson LÅ, Berhanu D. Quality of clinical assessment

For peer review only

For peer review only - http://bmjopen.bmj.com/site/about/guidelines.xhtml

and management of sick children by Health Extension Workers in four regions of Ethiopia: A cross-sectional survey. PLoS One. 2020;15(9):e0239361.

18. Getachew T, Mekonnen S, Yitayal M, Persson LÅ, Berhanu D. Health Extension Workers' diagnostic accuracy for common childhood illnesses in four regions of Ethiopia: a cross-sectional study. Acta Paediatr. 2019;108(11):2100–6.

19. Della B, Yemisrach BO, Atkure D, Abebe B, Ephrem Tekle L, Araya Abrha M, et al. Does a complex intervention targeting communities, health facilities and district health managers increase the utilisation of community- based child health services? A before and after study in intervention and comparison areas of Ethiopia. BMJ Open. 2020;10(e040868).

20. Persson LÅ. Bridging the quality chasm in maternal, newborn, and child healthcare in low- and middle-income countries. PLoS Med. 2017;14(12):12–4.

21. Berhanu D, Okwaraji YB, Belayneh AB, Lemango ET, Agonafer N, Birhanu BG, et al. Protocol for the evaluation of a complex intervention aiming at increased utilisation of primary child health services in Ethiopia: A before and after study in intervention and comparison areas. BMC Health Serv Res. 2020;20(1):1–12.

22. Duc D, Bergström A, Eriksson L, Selling K, Ha BTT, Wallin L. Response process and test-retest reliability of the Context Assessment for Community Health tool in Vietnam. Glob Health Action. 2016;9(1).

23. Yu DSF, Lee DTF, Woo J. Issues and challenges of instrument translation. West J Nurs Res. 2004;26(3):307–20.

24. Conrad F, Blair J. From impressions to data: Increasing the objectivity of cognitive interviews. Proc Sect Surv Res Methods, Annu Meet Stat Assoc. 1996;1(10):1–9.

25. WHO. Service Availability and Readiness Assessment (SARA) An annual monitoring system

For peer review only - http://bmjopen.bmj.com/site/about/guidelines.xhtml

for service delivery Reference Manual. 2013. WHO press, Geneva.

26. Gliem JA, Gliem RR. Midwest Research to Practice Conference in Adult, Continuing, and Community Education. 2003.

27. Coles E, Wells M, Maxwell M, Harris FM, Anderson J, Gray NM, et al. The influence of contextual factors on healthcare quality improvement initiatives: What works, for whom and in what setting? Protocol for a realist review. Syst Rev. 2017;6(1):1–10.

28. Kaplan HC, Brady PW, Dritz MC, Hooper DK, Linam WM, Froehle CM, et al. The influence of context on quality improvement success in health care: A systematic review of the literature. Milbank Q. 2010;88(4):500–59.

29. Mocumbi S, McKee K, Munguambe K, Chiau R, Högberg U, Hanson C, et al. Ready to deliver maternal and newborn care? Health providers' perceptions of their work context in rural Mozambique. Glob Health Action. 2018;11(1).

30. Datiko DG, Bunte EM, Birrie GB, Kea AZ, Steege R, Taegtmeyer M, et al. Community participation and maternal health service utilization: lessons from the health extension programme in rural southern Ethiopia. J Glob Heal Reports. 2019;3:1–12.

31. Kok MC, Kea AZ, Datiko DG, Broerse JEW, Dieleman M, Taegtmeyer M, et al. A qualitative assessment of health extension workers' relationships with the community and health sector in Ethiopia: Opportunities for enhancing maternal health performance. Hum Resour Health. 2015;13(1):1–12.

32. Mpembeni RNM, Bhatnagar A, LeFevre A, Chitama D, Urassa DP, Kilewo C, et al. Motivation and satisfaction among community health workers in Morogoro Region, Tanzania: Nuanced needs and varied ambitions. Hum Resour Health. 2015;13(1):1–10.

33. Jigssa HA, Desta BF, Tilahun HA, McCutcheon J, Berman P. Factors contributing to

For peer review only

motivation of volunteer community health workers in Ethiopia: The case of four woredas (districts) in Oromia and Tigray regions. Hum Resour Health. 2018;16(1):1–11.

34. Pourtaleb A, Jafari M, Seyedin H, Akhavan Behbahani A. New insight into the informal patients' payments on the evidence of literature: A systematic review study. BMC Health Serv Res. 2020;20(1):1–11.

35. Stringhini S, Thomas S, Bidwell P, Mtui T, Mwisongo A. Understanding informal payments in health care: Motivation of health workers in Tanzania. Hum Resour Health. 2009;7:1–9.

36. Leslie HH, Spiegelman D, Zhou X, Kruk ME. Service readiness of health facilities in Bangladesh, Haiti, Kenya, Malawi, Namibia, Nepal, Rwanda, Senegal, Uganda and the United Republic of Tanzania. Bull World Health Organ. 2017;95:738–48.

37. Acharya K, Paudel YR. General health service readiness and its association with the facility level indicators among primary health care centers and hospitals in Nepal. J Glob Heal Reports. 2019;3.

38. Okwaraji YB, Hill Z, Defar A, Berhanu D, Wolassa D, Persson LÅ, et al. Implementation of the 'optimising the health extension program' intervention in ethiopia: A process evaluation using mixed methods. Int J Environ Res Public Health. 2020;17(16):1–20.

39. Mahdavi M, Vissers J, Elkhuizen S, Van Dijk M, Vanhala A, Karampli E, et al. The relationship between context, structure, and processes with outcomes of 6 regional diabetes networks in Europe. PLoS One. 2018;13(2):1–17.

40. Kringos DS, Sunol R, Wagner C, Mannion R, Michel P, Klazinga NS, et al. The influence of context on the effectiveness of hospital quality improvement strategies: a review of systematic reviews. BMC Health Serv Res. 2015;15(277).

For peer review only

Data availability statement

Data are available up on request. Request for data can be made to Della Berhanu (della.berhanu@lshtm.ac.uk). Data sharing policy has been developed. All requests will be reviewed by data sharing committee and if granted, data will be shared without any identifiers.

Acknowledgements

The authors would like to thank the field teams that were involved in the data collection as well as the government official that facilitated the administration of the surveys. The authors would also like to thank the study participants who agreed to give their time to participate in the study.

Contributors

TG, SMA, MY, LAP, and DB conceptualized the design of the study. TG analysed the data; TG, SMA, MY, AB, LAP, and DB provided review of the methodology and interpret the results; All authors contributed to the writing of this paper and all have read and approved the final manuscript.

Funding

This project was funded by Bill & Melinda Gates Foundation (INV-009691). The funder had no role in study design, data collection, management, analysis, or interpretation of results.

Conflict of interest

None.

Patient and public involvement

Patients or the public were not involved in the design or conduct, or reporting or dissemination plans of this research.

Ethical considerations

For peer review only - http://bmjopen.bmj.com/site/about/guidelines.xhtml

Ethical approvals were obtained from the University of Gondar (Ref O/V/P/RCS/05/371/2018), the Ethiopian Public Health Institute (Ref 613/52), and the London School of Hygiene and Tropical Medicine (Ref 16117). Information sheets were translated into the local languages Amharic, Oromiffaa, and Tigrigna and read to obtain written informed consent.

Figure legend

Figure 1: Percent agreement to dimensions in the Context Assessment for Community Health tool. Health extension workers in four Ethiopian regions, 2018 (N=152).

Figure 2: Percentage of health posts with tracer items available to provide general service in four regions of Ethiopia, 2018 (N=152)

For peer review only

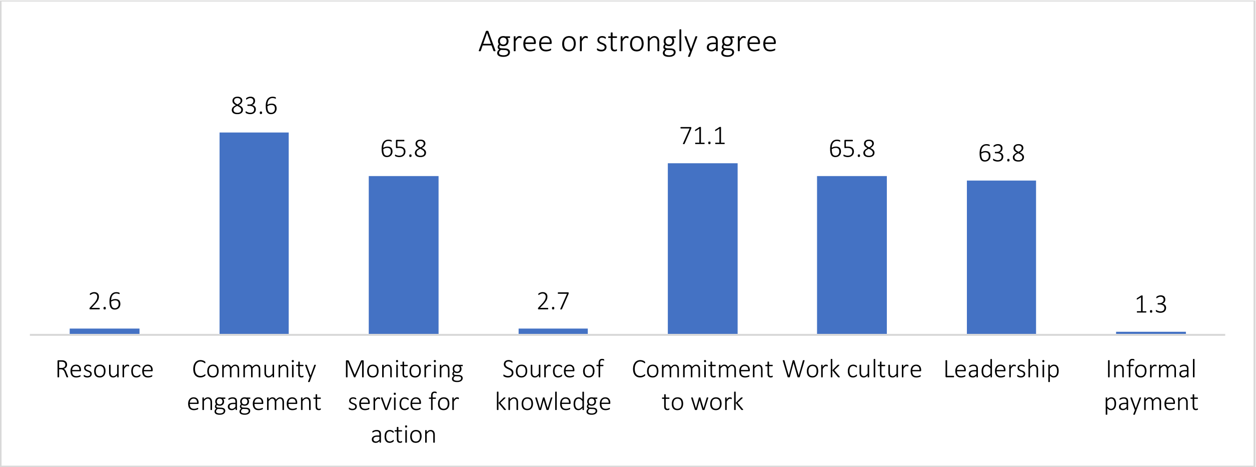

Figure 1: Percentage of average score for items of the context assessment for community health workers tool in four regions of Ethiopia, 2018 (N=152).

For peer review only

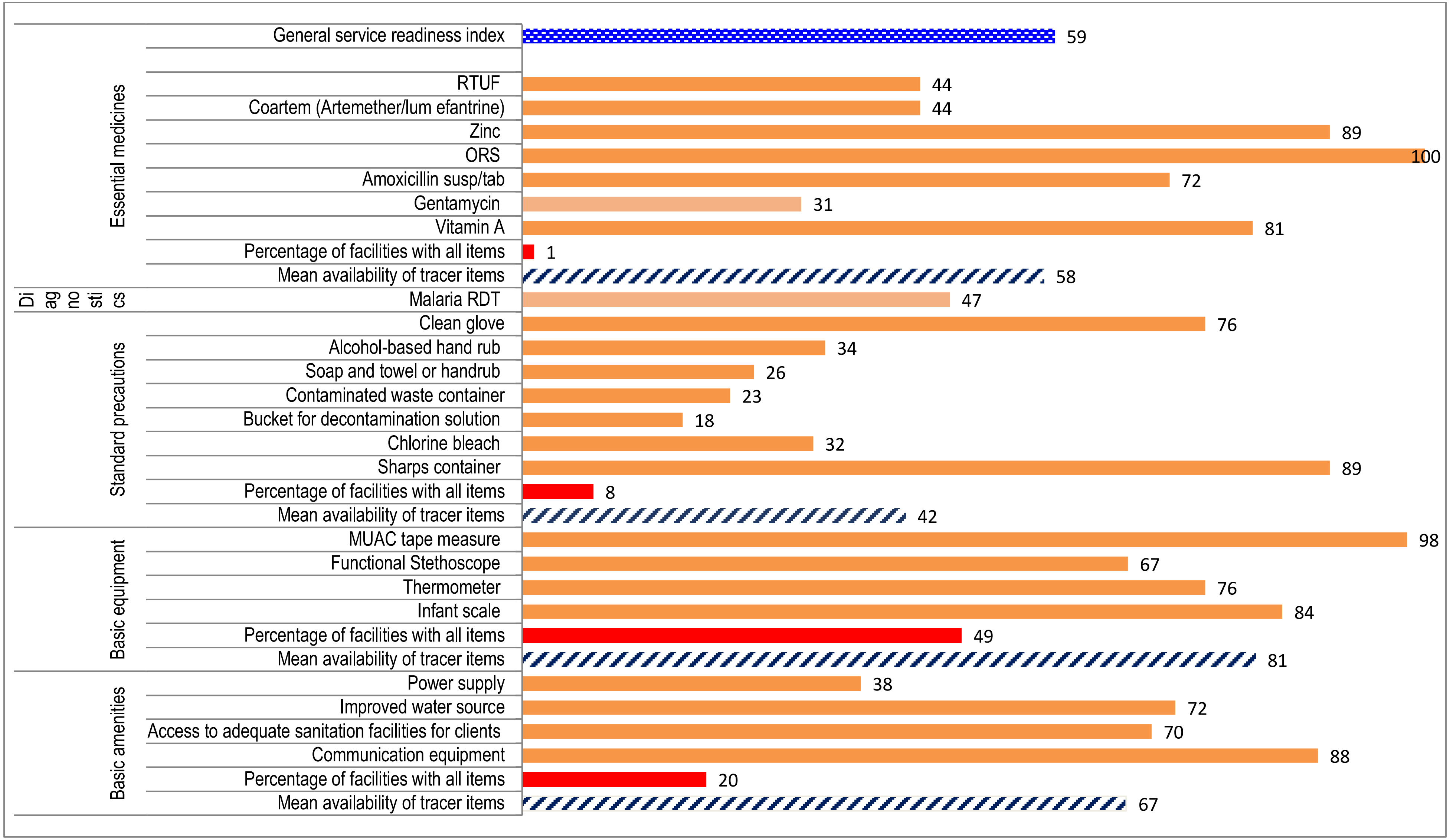

Figure 2: Percentage of health posts with tracer items available to provide general service in four regions of Ethiopia, 2018 (N=152)

Supplemental Table S1. Association between context dimensions and interventions and comparison areas in four regions of Ethiopia, 2018 (N=152).

Dimensions		Comparison (N=74)	Intervention (N=78)	Fisher's exact test
Resource	Agree	2.7	2.6	1
	Disagree	97.3	97.4	
Community engagement	Agree	78.4	88.5	0.125
	Disagree	21.6	11.5	
Monitoring service for action	Agree	58.1	73.1	0.061
	Disagree	41.9	26.9	
Source of knowledge	Agree	2.7	2.7	1
	Disagree	97.3	97.3	
Commitment to work	Agree	66.2	75.6	0.215
	Disagree	33.8	24.4	
Work culture	Agree	64.9	66.7	0.865
	Disagree	35.1	33.3	
Leadership	Agree	60.8	66.7	
	Disagree	39.2	33.3	0.501
Informal payment	Agree	1.4	1.3	1
	Disagree	98.7	98.7	

For peer review only

STROBE Statement—Checklist of items that should be included in reports of ***cross-sectional studies***

	Item No	Recommendation	Reported	Page number
**Title and abstract**	1	(*a*) Indicate the study's design with a commonly used term in the title or the abstract	✓	1
		(*b*) Provide in the abstract an informative and balanced summary of what was done and what was found	✓	2
**Introduction**				
Background/rationale	2	Explain the scientific background and rationale for the investigation being reported	✓	4-5
Objectives	3	State specific objectives, including any prespecified hypotheses	✓	5
**Methods**				
Study design	4	Present key elements of study design early in the paper	✓	5
Setting	5	Describe the setting, locations, and relevant dates, including periods of recruitment, exposure, follow-up, and data collection	✓	5
Participants	6	(*a*) Give the eligibility criteria, and the sources and methods of selection of participants	✓	6
Variables	7	Clearly define all outcomes, exposures, predictors, potential confounders, and effect modifiers. Give diagnostic criteria, if applicable	✓	6-7
Data sources/ measurement	8*	For each variable of interest, give sources of data and details of methods of assessment (measurement). Describe comparability of assessment methods if there is more than one group	✓	*8*
Bias	9	Describe any efforts to address potential sources of bias	✓	8
Study size	10	Explain how the study size was arrived at	✓	6
Quantitative variables	11	Explain how quantitative variables were handled in the analyses. If applicable, describe which groupings were chosen and why	✓	6-7
Statistical methods	12	(*a*) Describe all statistical methods, including those used to control for confounding	✓	8
		(*b*) Describe any methods used to examine subgroups and interactions		
		(*c*) Explain how missing data were addressed		
		(*d*) If applicable, describe analytical methods taking account of sampling strategy		
		(*e*) Describe any sensitivity analyses		
**Results**				
Participants	13*	(a) Report numbers of individuals at each stage of study—eg numbers potentially eligible, examined for eligibility, confirmed eligible, included in the study, completing follow-up, and analysed	✓	9
		(b) Give reasons for non-participation at each stage	✓	9
		(c) Consider use of a flow diagram		
Descriptive data	14*	(a) Give characteristics of study participants (eg	✓	9

For peer review only

		demographic, clinical, social) and information on exposures and potential confounders		
		(b) Indicate number of participants with missing data for each variable of interest		
Outcome data	15*	Report numbers of outcome events or summary measures	✓	9-10
Main results	16	(*a*) Give unadjusted estimates and, if applicable, confounder-adjusted estimates and their precision (eg, 95% confidence interval). Make clear which confounders were adjusted for and why they were included	✓	10-12
		(*b*) Report category boundaries when continuous variables were categorized		
		(*c*) If relevant, consider translating estimates of relative risk into absolute risk for a meaningful time period		
Other analyses	17	Report other analyses done—eg analyses of subgroups and interactions, and sensitivity analyses		
**Discussion**				
Key results	18	Summarise key results with reference to study objectives	✓	13
Limitations	19	Discuss limitations of the study, taking into account sources of potential bias or imprecision. Discuss both direction and magnitude of any potential bias	✓	15
Interpretation	20	Give a cautious overall interpretation of results considering objectives, limitations, multiplicity of analyses, results from similar studies, and other relevant evidence	✓	13-14
Generalisability	21	Discuss the generalisability (external validity) of the study results	✓	15
**Other information**				
Funding	22	Give the source of funding and the role of the funders for the present study and, if applicable, for the original study on which the present article is based	✓	22

*Give information separately for exposed and unexposed groups.

Note: An Explanation and Elaboration article discusses each checklist item and gives methodological background and published examples of transparent reporting. The STROBE checklist is best used in conjunction with this article (freely available on the Web sites of PLoS Medicine at http://www.plosmedicine.org/, Annals of Internal Medicine at http://www.annals.org/, and Epidemiology at http://www.epidem.com/). Information on the STROBE Initiative is available at www.strobe-statement.org.

For peer review only

BMJ Open

Health extension workers' perceived health system context and health post preparedness to provide services: A cross-sectional study in four Ethiopian regions

Journal:	*BMJ Open*
Manuscript ID	bmjopen-2020-048517.R1
Article Type:	Original research
Date Submitted by the Author:	24-Apr-2021
Complete List of Authors:	Getachew, Theodros ; Ethiopian Public Health Institute; University of Gondar College of Medicine and Health Sciences, Institute of Public Health<br>Abebe, Solomon; University of Gondar College of Medicine and Health Sciences, Institute of Public Health<br>Yitayal, Mezgebu ; University of Gondar, Health Services Management and Health Economics<br>Bergström, Anna; Uppsala Universitet<br>Persson, Lars; London School of Hygiene & Tropical Medicine; Ethiopian Public Health Institute<br>Berhanu, Della; London School of Hygiene & Tropical Medicine, Disease Control; Ethiopian Public Health Institute
**Primary Subject Heading**:	Health services research
Secondary Subject Heading:	Public health
Keywords:	Quality in health care < HEALTH SERVICES ADMINISTRATION & MANAGEMENT, Organisation of health services < HEALTH SERVICES ADMINISTRATION & MANAGEMENT, International health services < HEALTH SERVICES ADMINISTRATION & MANAGEMENT

SCHOLARONE™
Manuscripts

I, the Submitting Author has the right to grant and does grant on behalf of all authors of the Work (as defined in the below author licence), an exclusive licence and/or a non-exclusive licence for contributions from authors who are: i) UK Crown employees; ii) where BMJ has agreed a CC-BY licence shall apply, and/or iii) in accordance with the terms applicable for US Federal Government officers or employees acting as part of their official duties; on a worldwide, perpetual, irrevocable, royalty-free basis to BMJ Publishing Group Ltd ("BMJ") its licensees and where the relevant Journal is co-owned by BMJ to the co-owners of the Journal, to publish the Work in this journal and any other BMJ products and to exploit all rights, as set out in our licence.

The Submitting Author accepts and understands that any supply made under these terms is made by BMJ to the Submitting Author unless you are acting as an employee on behalf of your employer or a postgraduate student of an affiliated institution which is paying any applicable article publishing charge ("APC") for Open Access articles. Where the Submitting Author wishes to make the Work available on an Open Access basis (and intends to pay the relevant APC), the terms of reuse of such Open Access shall be governed by a Creative Commons licence – details of these licences and which Creative Commons licence will apply to this Work are set out in our licence referred to above.

Other than as permitted in any relevant BMJ Author's Self Archiving Policies, I confirm this Work has not been accepted for publication elsewhere, is not being considered for publication elsewhere and does not duplicate material already published. I confirm all authors consent to publication of this Work and authorise the granting of this licence.

For peer review only

Health extension workers' perceived health system context and health post preparedness to provide services: A cross-sectional study in four Ethiopian regions

Theodros Getachew[1,2], Solomon Mekonnen Abebe[2], Mezgebu Yitayal[2], Anna Bergström[3], Lars Åke Persson[1, 4], Della Berhanu[1, 4]

1. Health System and Reproductive Health Research Directorate, Ethiopian Public Health Institute, Addis Ababa, Ethiopia
2. College of Medicine and Health Sciences, Institute of Public Health, University of Gondar, Gondar, Ethiopia
3. Uppsala Global Health Research on Implementation and Sustainability (UGHRIS), Department of Women and Children Health, Uppsala University, Uppsala, Sweden
4. Department of Disease Control, London School of Hygiene & Tropical Medicine, Keppel Street, London, United Kingdom

Corresponding author: Theodros Getachew

Email: tedi.getachew@yahoo.com

For peer review only

Word count: 3,096

ABSTRACT

Objective: The health system context influences the implementation of evidence-based practices and quality of health care services. Ethiopia aims at reaching universal health coverage but faces low primary care utilisation and substandard quality-of-care. We assessed the health extension workers' perceived context and the preparedness of health posts to provide services.

Setting: This study was part of evaluating a complex intervention in 52 districts of four regions of Ethiopia. This paper used the endline data collected from December 2018 to February 2019.

Participants: A total of 152 health posts and health extension workers serving selected enumeration areas were included.

Outcome measures: We used the Context Assessment for Community Health (COACH) tool and the Service Availability and Readiness Assessment (SARA) tool.

Results: Internal reliability of COACH was satisfactory. The dimensions *community engagement*, *work culture*, *commitment to work*, and *leadership* all scored high (mean 3.75-4.01 on a 1-5 scale), while *organizational resources, sources of knowledge,* and *informal payments* scored low (1.78-2.71). The general service readiness index was 59%. On average, 67% of the health posts had basic amenities to provide services, 81% had basic equipment, 42% had standard precautions for infection prevention, 47% had test capacity for malaria, and 58% had essential medicines.

Conclusion: The health extension workers had a good relationship with the local community, used data for planning, were highly committed to their work with positive perceptions of their work culture, a relatively positive attitude regarding their leaders, and reported no corruption or informal payments. In contrast, they had insufficient sources of information and a severe lack of resources. The health post preparedness confirmed the low level of resources and preparedness for services. These findings suggest a significant potential contribution by health extension workers to Ethiopia's primary health care, provided that they receive improved support, including new information and essential resources.

Key words: Health system context, facility preparedness, health extension workers

For peer review only

Strengths and limitations of this study

- This study was the first assessment of Ethiopian front-line primary healthcare workers' perceived health system context and the facility preparedness for services.
- Understanding the health system context increases the likelihood of successful implementation of evidence-based practices.
- The Context Assessment for Community Health (COACH) tool has been validated in a range of other low-income countries and was found to have satisfactory internal reliability when translated into three Ethiopian languages.
- Although precautions were taken to obtain valid responses from the interviewed health extension workers, we cannot exclude the risk of respondents' bias.
- The sample represented 52 districts in four Ethiopian regions that participated in a child health services study, and inferences cannot be drawn to the whole country.

For peer review only

For peer review only - http://bmjopen.bmj.com/site/about/guidelines.xhtml

INTRODUCTION

The health system context is essential for new interventions and quality-of-care [1]. Health care of poor quality contributes to the high mortality in low- and middle-income countries [2, 3]. These quality problems have multiple causes, for example, lack of resources and suboptimal interaction between health care providers and clients [4]. Thus, there is a growing understanding that the health system context matters for efforts to improve health services quality [5, 6]. However, we lack consensus on the definition, operationalisation, and methods to study context [1].

Therefore, we need systematic ways of assessing the context in which health workers are active [7]. The most frequently used tools and information systems provide structural information, for example, the Service Availability and Readiness Assessment (SARA) tool [8]. Mapping the facility preparedness sets the scene, but such assessments are poorly associated with the quality of services provided [9]. A meta-review showed that access to information, community engagement, leadership, regulations and standards, organisational capacity, models of care, communication, and work satisfaction are essential for implementing new interventions and quality-of-care [6, 10]. The Context Assessment for Community Health (COACH) tool was developed and validated in low- and middle-income countries and included many of the context dimensions mentioned above [11].

In Ethiopia, primary-level services for under-five children are provided through the health extension programme [12, 13]. This programme is a community-based strategy to expand access to basic health promotion, disease prevention, and selected curative health services [14]. The programme is operated by the health extension workers at the community level [15]. Two female community health workers, known as health extension workers, provide preventative and curative services for a population of 5000. They offer static services from health posts as well as outreach services within the community [16]. The health extension workers are recruited from the community they serve and deployed to

For peer review only - http://bmjopen.bmj.com/site/about/guidelines.xhtml

service after a 1-year formal pre-service training provided after completing 10th grade of formal education [14]. Five health posts, their referral health centre, and a primary district-level hospital comprise the primary health care unit [17]. Health posts are the most peripheral units, providing mainly preventive care and selected curative services [18]. Despite the successful implementation of the health extension program, the program is currently facing challenges that remain to be addressed. These challenges are related to the utilisation and quality of services offered by the health extension workers and their working and living conditions [19].

The Ethiopian Ministry of Health aims to increase the primary health care services' access and quality through reforms and new initiatives [20, 21]. One such effort was the Optimizing the Health Extension Program intervention to increase the quality and utilisation of health services for under-five children. As part of the evaluation of that intervention, we have shown that health extension workers did not follow the clinical guidelines for assessing and managing sick children with common illnesses [22]. Their ability to classify childhood illnesses was also low [23]. Unfortunately, the intervention, which included community engagement, training, supportive supervision, and performance reviews of health workers, neither increased care-seeking for sick children [24] nor improved the classification of childhood illnesses by these primary health care workers [25]. The failure of such an intervention could, at least partly, be attributed to the context of the health extension workers. There is a need for accurate measurements that reflect the health system context in which care is provided to patients and populations [26]. Therefore, we aimed to assess the health extension workers' perceived health system context and the health posts' service readiness in four Ethiopian regions.

METHODS

Study setting and design

For peer review only

For peer review only - http://bmjopen.bmj.com/site/about/guidelines.xhtml

This study was part of a large project, which assessed a complex intervention's effectiveness to increase care-seeking for children under five years. This intervention had three components: community engagement, capacity building, and ownership and accountability of child health services. The assessment was done in 52 districts of four regions (Amhara, Tigray, Oromia, Southern Nations, Nationalities, and Peoples) of Ethiopia with baseline and end-line surveys conducted before and after the intervention. This paper used the endline data that was conducted from December 2018 to February 2019. The protocol and results of the evaluation have been published [24, 27].

Subjects

A total of 200 enumeration areas were selected to represent the selected districts in the end-line survey. Health posts serving these areas were included in the study, and their preparedness for services was assessed. One health extension worker at each health post was interviewed, and their perceived context was evaluated. We considered datasets with information from health posts as well as their respective health extension workers.

Study tools

Two tools were used at facility and provider levels. The provider-level tool aimed to assess the health extension workers' perceived context on the service delivery environment. The tool, labelled the Context Assessment for Community Health (COACH), has 49 items that measure eight dimensions of context (Table 1) [28]. The tool was developed in Bangladesh, Vietnam, Uganda, South Africa, and Nicaragua [11]. It also includes demographic questions on age, gender, professional qualification, health facility, and years working at the current facility. The tool items were measured on a five-point Likert scale ranging from 'strongly disagree' to 'strongly agree'. Items in *source of information* were measured for availability and frequency of use. The Brislin model [29] was used to translate the tool into Amharic, Oromiffaa, and Tigrigna, including forward translation, review of the translated tool,

For peer review only

For peer review only - http://bmjopen.bmj.com/site/about/guidelines.xhtml

backward translation, and comparison of the original and back-translated tools. The forward translation was done by a professional translator. The review, backward translation, and comparisons were done by a group of experts, including the study team. Conrad and Blair's taxonomy [30] was used to describe the problems that appeared in the translations. Accordingly, there were six lexical problems with difficulties in the meanings of words, one logical problem, and one inclusion or exclusion problem. All identified translation problems were possible to correct.

Table 1. Definitions of context assessment dimensions in the Context Assessment for Community Health tool.

Dimensions	Number of items	Definition
Resources	11	The availability of resources (staff, space, time, communication and transport, drugs, equipment and supplies, finance) that allows a unit to adapt successfully to internal and external pressures.
Community engagement	5	The mutual communication, deliberation and activities that occur between community members and units.
Monitoring services for action	5	The process of using data to assess group/team performance.
Sources of knowledge	5	The structures that facilitate the ability to access and use knowledge.
Commitment to work	3	The relative strength of an individual's identification with and involvement in a particular work organization.
Work culture	6	The way that 'we do things' in our organizations and work units. This includes culture of learning and change, and culture of responsibility.
Leadership	6	The actions of formal leaders in an organization (unit).
Informal payment	8	Payments to individuals, which are made outside official payment channels including nepotism, and accountability.

The facility tool aimed at collecting information on the overall facility-level preparedness to provide child health services (Table 2), which was based on the World Health Organization Service Availability and Readiness Assessment reference manual [31]. The tool was translated into three local languages (Amharic, Oromiffaa and Tigrigna), pretested and amended.

For peer review only - http://bmjopen.bmj.com/site/about/guidelines.xhtml

Table 2. Domains and their items used to construct the health post service availability and readiness.

Domain	Items
Basic amenities	Communication equipment
	Access to adequate sanitation facilities for clients
	Improved water source
	Power supply
Basic equipment	Infant scale
	Thermometer
	Functional stethoscope
	Mid-upper arm circumference tape measure
Standard precautions	Sharps container
	Chlorine bleach
	Bucket for decontamination solution
	Contaminated waste container
	Soap and towel or hand rub
	Alcohol-based hand rub
	Clean glove
Diagnostics	Malaria rapid diagnostic test
Essential medicines	Vitamin A
	Gentamycin
	Amoxicillin susp/tab
	Oral rehydration solution
	Zinc
	Coartem (Artemether Lumefantrine)
	Ready-to-use therapeutic food

Measurements

We judged the COACH tool's internal consistency with Cronbach alpha [32] that expressed if items in the instrument's different dimensions measured the same thing. Descriptive statistics were used to assess the health extension workers' agreement to the items and dimensions. All items except for source of knowledge were measured on a scale of 1-5, where the scores for item 48 and 49 were reversed to measure in the same direction as other items. The overall agreement was a calculated value drawn by multiplying the number of items in the dimension by four, which was coded as agreement. An individual was considered to agree if her score was above the calculated value.

For peer review only

For peer review only - http://bmjopen.bmj.com/site/about/guidelines.xhtml

The general health service readiness score was a composite summary measure calculated by combining information from the five general service readiness domains: basic amenities, standard precautions for infection prevention, basic equipment, diagnostics, and essential medicines [31]. For each domain, the average availability of tracer items was revealed as the domain score. Each dimension's mean score was computed to assess the average responses to the included items in the dimension. The analysis was performed using STATA 14.2 statistical package (Stata Corp LP, College Station, TX, USA).

Patient and public involvement

Patients or the public were not involved in the design or conduct, or reporting or dissemination plans of this research.

RESULTS

Of the 200 enumeration areas, 20 were not included due to local unrest. The remaining 180 enumeration areas were served by 165 health posts. A total of 165 health posts were assessed, and 154 health extension workers were available for interview. Eleven health posts did not have data on their respective health extension workers and two health extension workers were interviewed without their respective health post data. After merging the two datasets, 152 health post and health extension worker data were available for analysis.

Perceived context

Table 3 presents the average interitem correlation and the Cronbach Alpha coefficients for the eight context dimensions. Almost all dimensions exceeded the commonly accepted standard for satisfactory internal reliability (0.70) for new scales (α range = 0.51 to 0.89). One dimension (*source of knowledge*, α = 0.51) did not meet this standard. The average inter-item correlation ranged from 0.17 to 0.59. The ideal range of average inter-item correlation is 0.15 to 0.50; less than 0.15 indicates that items are not

For peer review only

For peer review only - http://bmjopen.bmj.com/site/about/guidelines.xhtml

well correlated and don't measure the same idea very well. More than 0.50 means that items are close, almost repetitive.

The mean scores of the COACH dimensions on a scale of 1-5 are presented in table 3. The dimensions *community engagement*, *work culture*, *commitment to work*, and *leadership* all scored high (mean 3.75-4.01 on the 1-5 scale), while *organizational resources, sources of knowledge,* and *informal payments* scored low (1.78-2.71). These findings indicate that the health extension workers neither perceived themselves as having sufficient resources to conduct their work nor to have access to new knowledge.

Table 3. Summary of perceived context of health extension workers and the internal consistency of the Context Assessment for Community Health tool. Survey in four Ethiopian regions, 2018. (N=152)

Dimensions	Number of items	Mean (SD)	Cronbach alpha	Average interitem correlation
Resources	11	2.60 (0.60)	0.7620	0.2255
Community engagement	5	4.01 (0.58)	0.8813	0.5975
Monitoring services for action	5	3.75 (0.70)	0.8678	0.5676
Sources of knowledge	5	2.71 (0.79)	0.5053	0.1696
Commitment to work	3	3.79 (0.79)	0.7976	0.5677
Work culture	6	3.89 (0.51)	0.7683	0.3559
Leadership	6	3.79 (0.60)	0.8771	0.5432
Informal payment	8	1.78 (0.56)	0.8427	0.4011

Table 4 and 5 depicts the percentage of each item included in the eight context dimensions. Most of the health extension workers reported disagreement on the availability of financial resources. They also disagreed to having access to communication and transport.

Table 4. Percentage of items and dimensions of the Context Assessment for Community Health tool in four Ethiopian regions, 2018 (N=152).

Resource	Disagree	Neutral	Agree
1. My unit has enough workers with the right training and skills to do everything that needs to be done.	52	3	45
2. My unit has enough workers with the right training and skills to do their job in the best possible way.	52	2	46
3. My unit has enough space to provide healthcare services.	51	2	47
4. My unit has access to the transport and fuel that are needed to provide healthcare services.	88	0	13

For peer review only - http://bmjopen.bmj.com/site/about/guidelines.xhtml
For peer review only

5. My unit has access to the communication tools (e.g., telephone or radio) that are needed to provide healthcare services.	84	2	14
6. My unit has enough medicine to provide healthcare services.	48	2	50
7. My unit has enough functional equipment, such as a thermometer and blood pressure cuff, to provide healthcare services.	49	4	47
8. My unit has enough disposable medical equipment, such as syringes, gloves and needles, to provide healthcare services.	30	0	70
9. If the workload increases, my unit can get additional resources such as medicine and equipment.	45	2	53
10. My unit receives money according to an established financial plan.	84	2	14
11. My unit has money that we can decide how to use.	91	3	6
Community engagement			
12. In my unit we ask community members what they think about the healthcare services that we provide.	7	0	93
13. In my unit we listen to what community members think about the healthcare services we provide.	4	1	95
14. In my unit we have meetings with community members to discuss health matters.	5	1	93
15. In my unit we encourage community members to contribute to improving the health of the community.	3	1	96
16. In my unit we encourage other organizations to contribute to improving the health of the community.	11	0	89
Monitoring services for action			
17. I receive regular updates about my unit's performance based on information/data collected from our unit.	14	3	84
18. My unit discusses information/data from our unit in a regular, formal way, such as in regularly scheduled meetings.	11	7	82
19. My unit regularly uses unit information/data to make plans for improving its healthcare services.	13	4	84
20. My unit regularly monitors its work by comparing it with the unit's action plans.	13	5	83
21. My unit regularly compares its work with national or other guidelines.	16	2	82
Commitment to work			
27. I am proud to work in this unit.	21	3	76
28. I am satisfied to work in this unit.	16	5	80
29. I feel encouraged to do my very best at work.	7	3	89
Work culture			
30. My unit is willing to use new healthcare practices such as guidelines and recommendations.	4	1	95
31. My unit helps me to improve and develop my skills.	28	2	70
32. I am encouraged to seek new information on healthcare practices.	20	3	78
33. My unit works for the good of the clients and puts their needs first.	6	1	93
34. Members of the unit feel personally responsible for improving healthcare services.	6	0	94
35. Members of the unit approach clients with respect.	2	2	96
Leadership			
36. I trust the unit leader.	7	3	91
37. The leader handles stressful situations calmly.	12	4	84
38. The leader actively listens, acknowledges, and then responds to requests and concerns.	11	4	85
39. The leader effectively resolves any conflicts that arise.	14	5	82
40. The leader encourages the introduction of new ideas and practices.	13	4	83
41. The leader makes things happen.	11	5	85
Informal payment			
42. Clients must always give informal payment to health workers to access healthcare services.	97	1	3

For peer review only

43. Clients are treated more quickly if they make informal payments to health workers.	98	0	2
44. Medicines or equipment that should be available for free to clients have been sold in my unit.	97	1	3
45. Health workers are sometimes absent from work earning money at other places.	97	1	2
46. Health workers in my unit give healthcare services to friends and family first.	95	1	3
47. Health workers in my unit give jobs or other benefits to friends and family first.	97	1	3
48. Efforts are made to stop clients from providing informal payment to get appropriate healthcare services.	21	7	72
49. Efforts are made to stop health workers from asking clients for informal payment.	21	8	71

Table 5. Percentage of items and dimensions in source of knowledge of the Context Assessment for Community Health tool in four Ethiopian regions, 2018 (N=152).

Sources of knowledge	Not available	Never, 0 times	Rarely, 1-5 times	Occasionally, 6-10 times	Frequently, 11-15 times	Almost always, 16 times or more
22. Clinical practice guidelines	15	4	18	16	16	32
23. Other printed material for work (e.g., textbooks, journals)	18	9	25	30	14	5
24. The Internet	68	25	5	1	0	1
25. Electronic decision support (e.g., mobile phone applications or other electronic devices to assist with care and decision-making)	56	30	3	5	5	2
26. In-service training/ workshops/courses	25	14	19	28	10	4

Figure 1 depicts the percentage of average scores for the context dimensions. Very few (2.6%) perceived their facility to have enough resources available to manage their work. Most respondents (83.6%) perceived that their facility had active communication with members of their communities. Sixty-six percent on average responded agreement for the *work culture* dimension, implying that they considered their *work culture* to support learning, change, and responsibility. A very high proportion of respondents (98.7%) regarded *informal payment* for health workers not to be acceptable in their facility.

Figure 1: Percent agreement to dimensions in the Context Assessment for Community Health tool. Health extension workers in four Ethiopian regions, 2018 (N=152).

For peer review only - http://bmjopen.bmj.com/site/about/guidelines.xhtml

There was no difference in context dimensions between intervention and comparison areas in the evaluation's end-line survey (all $p > 0.05$). The exact percentage for each item is found Supplemented (table S1).

General facility-level readiness

Figure 2 shows the general service readiness index and domain scores. The general service readiness index was 59%, implying that six in ten health posts were ready to provide child health services. On average, about two-thirds (67%) of health posts had basic amenities to provide services, 81% had basic equipment required, 42% had standard precautions for infection prevention, 47% had diagnostic test capacity for malaria RDT, and 58% had essential medicines. The basic equipment mean score was the highest across the five domains, and the diagnostic mean score index was the lowest.

Figure 2 also shows the percentage of health posts having all tracer items available to provide general child health services. Accordingly, only 1% of health posts had all essential medicines. Half of the health posts had all tracer items for basic equipment. Three in ten health posts had all items for basic amenities.

Figure 2: Percentage of health posts with tracer items available to provide general service in four regions of Ethiopia, 2018 (N=152)

DISCUSSION

We have described the Ethiopian health extension workers' perceived context and the health posts' preparedness to provide child health services. The health extension workers perceived that they had a good relationship with the local community. They were active in using data for planning and performance, were highly committed to their work and had positive perceptions of their work culture. They also had a relatively positive experience of their leaders and reported no corruption or informal payments. In contrast, they reported having insufficient information sources and a severe lack of

For peer review only - http://bmjopen.bmj.com/site/about/guidelines.xhtml

resources to perform their work. The latter was also reflected in the health post preparedness assessment, which overall was on a low level.

So far, there is no consensus on defining or assessing the health system context [33]. Several contextual factors are associated with quality improvement, like leadership, organizational culture, information system, and organizational structure. However, there are uncertainties regarding definitions and measurements [34]. Qualitative studies have contributed to the understanding of the health system context and quality of care. In this study, we quantified the perceived context and compared it with health post preparedness. The COACH tool was developed in five countries. Later, it has been used in Mozambique [35], and now in four different Ethiopian regions and three languages. Except for the sources of knowledge dimension, all other COACH tool dimensions showed good internal reliability. Items included in *source of knowledge* might not measure the same construct. The original validation process of this tool also indicated a low internal reliability of this dimension [11]. Some of the included items may not be relevant in certain contexts. We suggest this dimension with its five items for further evaluation.

The health extension workers lacked sources of new knowledge. Internet, e-health, or m-health applications were absent [35]. Importantly, their responses indicated that they lacked in-service training, workshops, and courses. Insufficient sources of knowledge could lead to inappropriate diagnosis and mismanagement, such as the irrational use of antibiotics. An earlier study conducted in the same study area indicated that the health extension workers' clinical assessment, classification, and management of sick children did not follow the clinical guidelines [22]. This low adherence could lead to misdiagnoses and a lack of potentially life-saving treatments. Capacity building could be achieved through refresher training, followed by supportive supervision.

For peer review only

For peer review only - http://bmjopen.bmj.com/site/about/guidelines.xhtml

The health extension workers reportedly had good contact with the community they served. This engagement could help to enhance the health extension workers' accountability and dedication. A study conducted in southern Ethiopia indicated that with focused training, guidance, and regular supportive supervision, the health extension workers enhanced in community participation [36]. A qualitative study in southern Ethiopia revealed that health extension workers' relationships with the community could be constrained due to inadequate support systems, trust, communication, and dialogue, as well as differing expectations [37]. A study conducted in six regions of Ethiopia indicated that there were challenges in work schedule and relationship with the community [38].

We also found that commitment to work was relatively good. A combination of financial and non-financial incentives is required to support motivation and satisfaction [39]. Non-financial incentives, such as creating career opportunities, may increase the motivation and retention of health extension workers [40]. Over the course of a week, the health extension workers spend their time at the health post (51%), in the community (37%) and elsewhere, unable to get information, (12%) [15]. This is an indication that they spend most of their time with the community that has a potential to enhance the interpersonal communication.

Informal payments were perceived to be very rare. Informal payments or various forms of corruption could have significant adverse effects on the health system, affecting patients and service providers [41]. A study in Tanzania showed that informal payments existed and were negatively associated with job satisfaction and motivation [42].

The health posts in the study area seemed to have moderate service readiness, especially basic amenities and equipment. However, health posts did not have the essential medicines to provide child care. These facilities are the first contact for primary care, which provides basic health and medical care close to the community, especially in rural populations [19]. These first-line services can

For peer review only

For peer review only - http://bmjopen.bmj.com/site/about/guidelines.xhtml

potentially respond to a range of health challenges in low-income countries [43], but only if proper attention is given to needs, such as essential medicines, in addition to infrastructure and basic equipment. To meet such requirements is vital for a resilient health system [44].

We have earlier shown that health extension workers' ability to classify childhood illnesses was low [23]. The evaluation of the Optimizing the Health Extension Program intervention's effectiveness showed no effect on the utilization of services for sick children [24]. The lack of effect could partly be attributed to delays, interruptions, and an overall short implementation period of a complex intervention. Complex interventions that aim to change health services and care-seeking for sick children may need an extended implementation period [45]. Lack of effect could also be due to some of these contextual factors necessary for improving quality.

The context in which the services are provided is essential for implementing changes or new programmes. However, contextual factors are generally not well understood. Before this study, no assessments of different aspects of the health system context have been done in Ethiopia. A study conducted in six European countries found that structure and process indicators explained more variability in client satisfaction than contextual factors [46]. A systematic review concluded that contextual factors might influence the effectiveness of quality improvement interventions [47].

We report the first study in Ethiopia of primary healthcare workers' perceived health system context. The Context Assessment for Community Health tool has been validated in a range of other low-income countries [11] and was also found to have satisfactory internal reliability when translated into three Ethiopian languages. Understanding context can identify factors that promote or hinder the implementation of evidence-based practices, increasing the likelihood of successful implementation.

Although precautions were taken to obtain valid responses from the interviewed health extension workers, the results could be susceptible to bias. The sample represented a large number of districts

For peer review only

For peer review only - http://bmjopen.bmj.com/site/about/guidelines.xhtml

in four Ethiopian regions that participated in a child health services study, but inferences cannot be drawn to the whole country.

CONCLUSION

The Ethiopian health extension workers' perceived context showed a severe lack of resources. They perceived a good relationship with the local community, used data for planning but lacked access to new knowledge. They were highly committed to work and had positive perceptions of their work culture and a relatively positive attitude regarding their leaders. There was no corruption or informal payments at their work sites. The internal consistency of the context assessment tool provided evidence of its ability to measure its different dimensions. This feature will allow for tailoring implementation strategies and assessing context as part of evaluations. The health extension workers' perceptions of sources of information and available resources were in line with the results of the health facility preparedness.

Contributors

TG, SMA, MY, LAP, and DB conceptualized the design of the study. TG analysed the data; TG, SMA, MY, AB, LAP, and DB provided review of the methodology and interpret the results; All authors contributed to the writing of this paper and all have read and approved the final manuscript.

Conflict of interest

None.

Funding

This project was funded by Bill & Melinda Gates Foundation (INV-009691). The funder had no role in study design, data collection, management, analysis, or interpretation of results.

Data availability statement

For peer review only - http://bmjopen.bmj.com/site/about/guidelines.xhtml

Data are available up on request. Request for data can be made to Della Berhanu (della.berhanu@lshtm.ac.uk). Data sharing policy has been developed. All requests will be reviewed by data sharing committee and if granted, data will be shared without any identifiers.

For peer review only

REFERENCES

1. Rogers L, De Brún A, McAuliffe E. Defining and assessing context in healthcare implementation studies: A systematic review. BMC Health Serv Res. 2020;20(1):1–24.

2. Kruk ME, Gage AD, Joseph NT, Danaei G, García-Saisó S, Salomon JA. Mortality due to low-quality health systems in the universal health coverage era: a systematic analysis of amenable deaths in 137 countries. Lancet. 2018 Nov 17;392(10160):2203–12.

3. Chou VB, Walker N, Kanyangarara M. Estimating the global impact of poor quality of care on maternal and neonatal outcomes in 81 low- And middle-income countries: A modeling study. PLoS Med. 2019;16(12).

4. Mosadeghrad AM. Factors affecting medical service quality. Iran J Public Health. 2014;43(2):210–20.

5. Mcdonald, Kathryn M, Ferguson M. Considering Context in Quality Improvement Interventions and Implementation : Concepts , Frameworks , and Application. Acadamic Pediatr. 2013;13(6):S45–53.

6. Akachi Y, Kruk ME. Quality of care : measuring a neglected driver of improved health. Bull World Health Organ. 2017;95:465–72.

7. Daivadanam M, Ingram M, Annerstedt KS, Parker G, Bobrow K, Dolovich L, et al. The role of context in implementation research for non-communicable diseases: Answering the "how-to" dilemma. PLoS One. 2019;14(4):1–22.

8. World Health Organization. Service Availability and Readiness Assessment (SARA): An annual monitoring system for service delivery Reference Manual. In Geneva: World Health Organization; 2013.

9. Leslie HH, Sun Z, Kruk ME. Association between infrastructure and observed quality of care in 4 healthcare services : A cross-sectional study of 4 , 300 facilities in 8 countries. PLOS Med.

For peer review only

2017;14(12):e1002464.

10. Nair M, Yoshida S, Lambrechts T, Boschi-Pinto C, Bose K, Mason EM, et al. Facilitators and barriers to quality of care in maternal, newborn and child health: A global situational analysis through metareview. BMJ Open. 2014;4(5).

11. Bergström A, Skeen S, Duc DM, Blandon EZ, Estabrooks C, Gustavsson P, et al. Health system context and implementation of evidence-based practices—development and validation of the Context Assessment for Community Health (COACH) tool for low- and middle-income settings. Implement Sci. 2015;10(1).

12. Gebrehiwot TG, Sebastian MS, Edin K, Goicolea I. The Health Extension Program and its association with change in utilization of selected maternal health services in tigray region, Ethiopia: A segmented linear regression analysis. PLoS One. 2015;10(7):1–15.

13. Yitayal M, Berhane Y, Worku A, Kebede Y. The community-based Health Extension Program significantly improved contraceptive utilization in West Gojjam Zone, Ethiopia. J Multidiscip Healthc. 2014;7:201–8.

14. Tilahun H, Fekadu B, Abdisa H, Canavan M, Linnander E, Bradley EH, et al. Ethiopia's health extension workers use of work time on duty: Time and motion study. Health Policy Plan. 2017;32(3):320–8.

15. Mangham-Jefferies L, Mathewos B, Russell J, Bekele A. How do health extension workers in Ethiopia allocate their time? Hum Resour Health. 2014 Dec 14;12(1):61.

16. Wang H, Tesfaye R, Ramana GN V, Chekagn CT. Ethiopia Health Extension Program: an institutionalized community approach for universal health coverage. 2016.

17. Yitayal M, Berhane Y, Worku A, Kebede Y. Health extension program factors , frequency of household visits and being model households , improved utilization of basic health services in

For peer review only

For peer review only - http://bmjopen.bmj.com/site/about/guidelines.xhtml

Ethiopia. BMC Health Serv Res [Internet]. 2014;14(1):1–9. Available from: BMC Health Services Research

18. Assebe LF, Belete WN, Alemayehu S, Asfaw E, Godana KT, Alemayehu YK, et al. Economic evaluation of Health Extension Program packages in Ethiopia. PLoS One [Internet]. 2021;16(2 February):1–16. Available from: http://dx.doi.org/10.1371/journal.pone.0246207

19. Assefa Y, Gelaw YA, Hill PS, Taye BW, Van Damme W. Community health extension program of Ethiopia, 2003-2018: Successes and challenges toward universal coverage for primary healthcare services. Global Health. 2019;15(1):1–11.

20. Argaw MD, Desta BF, Bele TA, Ayne AD. Improved performance of district health systems through implementing health center clinical and administrative standards in the Amhara region of Ethiopia. BMC Health Serv Res. 2019;19(1):1–13.

21. Ministry of Health. Ethiopian Health Sector Transformation Plan.2015/16 - 2019/20. Vol. 20, Federal Democratic Republic of Ethiopia Ministry of Health. 2015.

22. Daka DW, Wordofa MA, Woldie M, Persson LÅ, Berhanu D. Quality of clinical assessment and management of sick children by Health Extension Workers in four regions of Ethiopia: A cross-sectional survey. PLoS One. 2020;15(9):e0239361.

23. Getachew T, Mekonnen S, Yitayal M, Persson LÅ, Berhanu D. Health Extension Workers' diagnostic accuracy for common childhood illnesses in four regions of Ethiopia: a cross-sectional study. Acta Paediatr. 2019;108(11):2100–6.

24. Della B, Yemisrach BO, Atkure D, Abebe B, Ephrem Tekle L, Araya Abrha M, et al. Does a complex intervention targeting communities, health facilities and district health managers increase the utilisation of community- based child health services? A before and after study in intervention and comparison areas of Ethiopia. BMJ Open. 2020;10(e040868).

For peer review only - http://bmjopen.bmj.com/site/about/guidelines.xhtml

25. Getachew T, Abebe SM, Yitayal M, Persson LÅ, Berhanu D. Association between a complex community intervention and quality of health extension workers' performance to correctly classify common childhood illnesses in four regions of Ethiopia. PLoS One. 2021;16(3 March):1–13.

26. Persson LÅ. Bridging the quality chasm in maternal, newborn, and child healthcare in low- and middle-income countries. PLoS Med. 2017;14(12):12–4.

27. Berhanu D, Okwaraji YB, Belayneh AB, Lemango ET, Agonafer N, Birhanu BG, et al. Protocol for the evaluation of a complex intervention aiming at increased utilisation of primary child health services in Ethiopia: A before and after study in intervention and comparison areas. BMC Health Serv Res. 2020;20(1):1–12.

28. Duc D, Bergström A, Eriksson L, Selling K, Ha BTT, Wallin L. Response process and test-retest reliability of the Context Assessment for Community Health tool in Vietnam. Glob Health Action. 2016;9(1).

29. Yu DSF, Lee DTF, Woo J. Issues and challenges of instrument translation. West J Nurs Res [Internet]. 2004 Apr 1 [cited 2020 Oct 15];26(3):307–20. Available from: http://journals.sagepub.com/doi/10.1177/0193945903260554

30. Conrad F, Blair J. From impressions to data: Increasing the objectivity of cognitive interviews. Proc Sect Surv Res Methods, Annu Meet Stat Assoc. 1996;1(10):1–9.

31. WHO. Service Availability and Readiness Assessment (SARA) An annual monitoring system for service delivery Reference Manual. 2013.

32. Gliem JA, Gliem RR. Midwest Research to Practice Conference in Adult, Continuing, and Community Education. 2003.

33. Coles E, Wells M, Maxwell M, Harris FM, Anderson J, Gray NM, et al. The influence of

For peer review only

For peer review only - http://bmjopen.bmj.com/site/about/guidelines.xhtml

contextual factors on healthcare quality improvement initiatives: What works, for whom and in what setting? Protocol for a realist review. Syst Rev. 2017;6(1):1–10.

34. Kaplan HC, Brady PW, Dritz MC, Hooper DK, Linam WM, Froehle CM, et al. The influence of context on quality improvement success in health care: A systematic review of the literature. Milbank Q. 2010;88(4):500–59.

35. Mocumbi S, McKee K, Munguambe K, Chiau R, Högberg U, Hanson C, et al. Ready to deliver maternal and newborn care? Health providers' perceptions of their work context in rural Mozambique. Glob Health Action. 2018;11(1).

36. Datiko DG, Bunte EM, Birrie GB, Kea AZ, Steege R, Taegtmeyer M, et al. Community participation and maternal health service utilization: lessons from the health extension programme in rural southern Ethiopia. J Glob Heal Reports. 2019;3:1–12.

37. Kok MC, Kea AZ, Datiko DG, Broerse JEW, Dieleman M, Taegtmeyer M, et al. A qualitative assessment of health extension workers' relationships with the community and health sector in Ethiopia: Opportunities for enhancing maternal health performance. Hum Resour Health. 2015;13(1):1–12.

38. Teklehaimanot A, Kitaw Y, G/Yohannes A, Girma S, Seyoum S, Desta S, et al. Study of the Working Conditions of Health Extension Workers in Ethiopia. Ethiop J Heal Dev. 2008 Apr 15;21(3).

39. Mpembeni RNM, Bhatnagar A, LeFevre A, Chitama D, Urassa DP, Kilewo C, et al. Motivation and satisfaction among community health workers in Morogoro Region, Tanzania: Nuanced needs and varied ambitions. Hum Resour Health. 2015;13(1):1–10.

40. Jigssa HA, Desta BF, Tilahun HA, McCutcheon J, Berman P. Factors contributing to motivation of volunteer community health workers in Ethiopia: The case of four woredas

For peer review only

(districts) in Oromia and Tigray regions. Hum Resour Health. 2018;16(1):1–11.

41. Pourtaleb A, Jafari M, Seyedin H, Akhavan Behbahani A. New insight into the informal patients' payments on the evidence of literature: A systematic review study. BMC Health Serv Res. 2020;20(1):1–11.

42. Stringhini S, Thomas S, Bidwell P, Mtui T, Mwisongo A. Understanding informal payments in health care: Motivation of health workers in Tanzania. Hum Resour Health. 2009;7:1–9.

43. Leslie HH, Spiegelman D, Zhou X, Kruk ME. Service readiness of health facilities in Bangladesh, Haiti, Kenya, Malawi, Namibia, Nepal, Rwanda, Senegal, Uganda and the United Republic of Tanzania. Bull World Health Organ. 2017;95:738–48.

44. Acharya K, Paudel YR. General health service readiness and its association with the facility level indicators among primary health care centers and hospitals in Nepal. J Glob Heal Reports. 2019;3.

45. Okwaraji YB, Hill Z, Defar A, Berhanu D, Wolassa D, Persson LÅ, et al. Implementation of the 'optimising the health extension program' intervention in ethiopia: A process evaluation using mixed methods. Int J Environ Res Public Health. 2020;17(16):1–20.

46. Mahdavi M, Vissers J, Elkhuizen S, Van Dijk M, Vanhala A, Karampli E, et al. The relationship between context, structure, and processes with outcomes of 6 regional diabetes networks in Europe. PLoS One. 2018;13(2):1–17.

47. Kringos DS, Sunol R, Wagner C, Mannion R, Michel P, Klazinga NS, et al. The influence of context on the effectiveness of hospital quality improvement strategies: a review of systematic reviews. BMC Health Serv Res. 2015;15(277).

For peer review only - http://bmjopen.bmj.com/site/about/guidelines.xhtml

Acknowledgements

The authors would like to thank the field teams that were involved in the data collection as well as the government official that facilitated the administration of the surveys. The authors would also like to thank the study participants who agreed to give their time to participate in the study.

Ethical considerations

Ethical approvals were obtained from the University of Gondar (Ref O/V/P/RCS/05/371/2018), the Ethiopian Public Health Institute (Ref 613/52), and the London School of Hygiene and Tropical Medicine (Ref 16117). Information sheets were translated into the local languages Amharic, Oromiffaa, and Tigrigna and read to obtain written informed consent.

Figure legend

Figure 1: Percent agreement to dimensions in the Context Assessment for Community Health tool. Health extension workers in four Ethiopian regions, 2018 (N=152).

Figure 2: Percentage of health posts with tracer items available to provide general service in four regions of Ethiopia, 2018 (N=152)

For peer review only

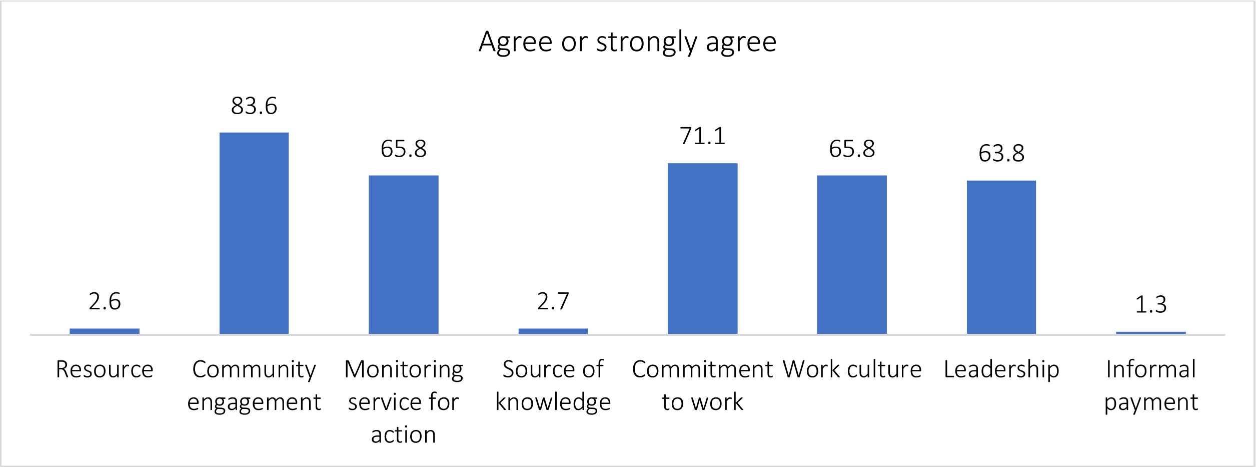

Figure 1: Percentage of average score for items of the context assessment for community health workers tool in four regions of Ethiopia, 2018 (N=152).

For peer review only

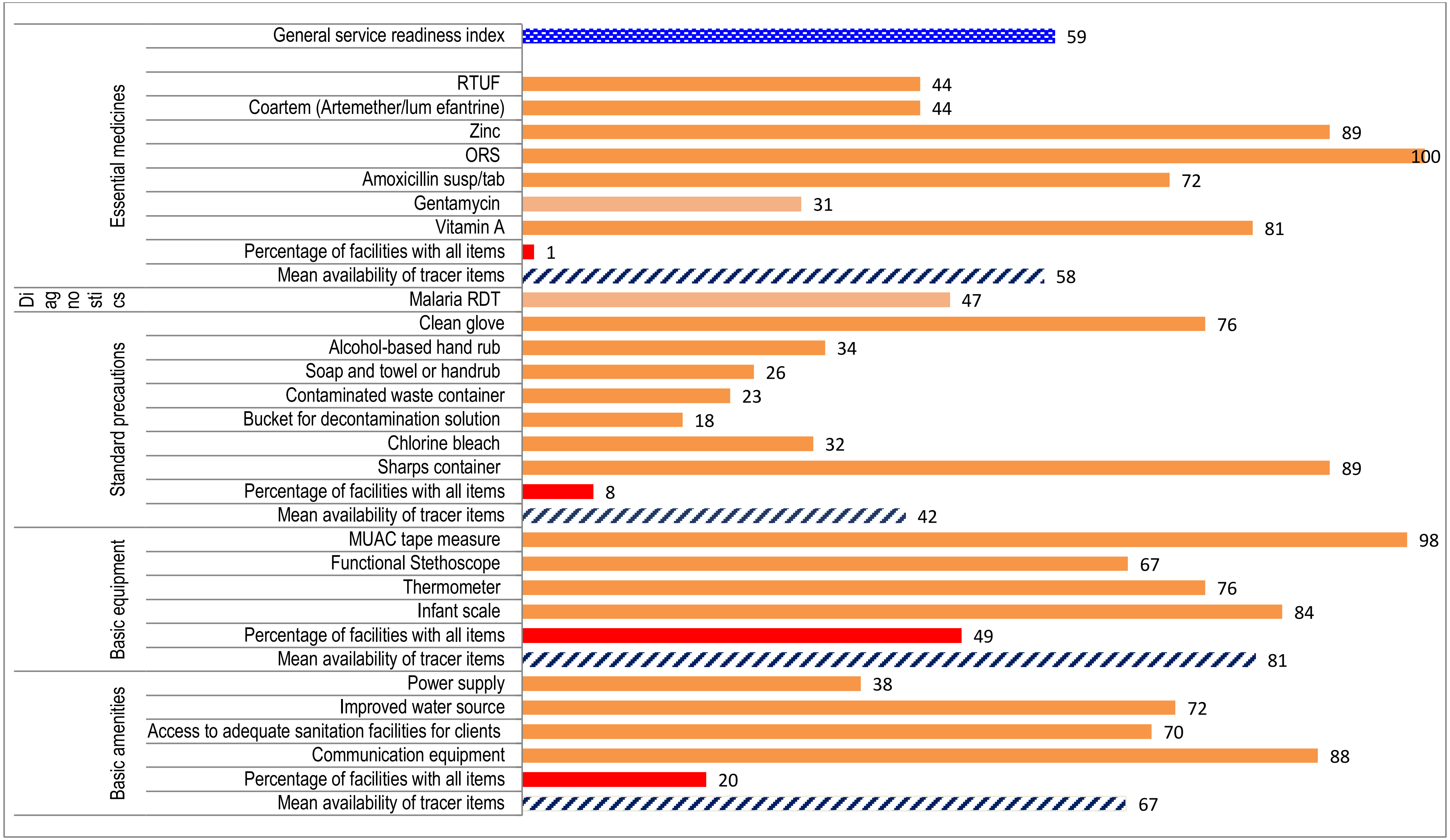

Figure 2: Percentage of health posts with tracer items available to provide general service in four regions of Ethiopia, 2018 (N=152)

Supplemental Table S1. Association between context dimensions and interventions and comparison areas in four regions of Ethiopia, 2018 (N=152).

Dimensions		Comparison (N=74)	Intervention (N=78)	Fisher's exact test
Resource	Agree	2.7	2.6	1
	Disagree	97.3	97.4	
Community engagement	Agree	78.4	88.5	0.125
	Disagree	21.6	11.5	
Monitoring service for action	Agree	58.1	73.1	0.061
	Disagree	41.9	26.9	
Source of knowledge	Agree	2.7	2.7	1
	Disagree	97.3	97.3	
Commitment to work	Agree	66.2	75.6	
	Disagree	33.8	24.4	0.215
Work culture	Agree	64.9	66.7	
	Disagree	35.1	33.3	0.865
Leadership	Agree	60.8	66.7	
	Disagree	39.2	33.3	0.501
Informal payment	Agree	1.4	1.3	1
	Disagree	98.7	98.7	

For peer review only

STROBE Statement—Checklist of items that should be included in reports of ***cross-sectional studies***

	Item No	Recommendation	Reported	Page number
**Title and abstract**	1	(*a*) Indicate the study's design with a commonly used term in the title or the abstract	✓	1
		(*b*) Provide in the abstract an informative and balanced summary of what was done and what was found	✓	2
**Introduction**				
Background/rationale	2	Explain the scientific background and rationale for the investigation being reported	✓	4-5
Objectives	3	State specific objectives, including any prespecified hypotheses	✓	5
**Methods**				
Study design	4	Present key elements of study design early in the paper	✓	5
Setting	5	Describe the setting, locations, and relevant dates, including periods of recruitment, exposure, follow-up, and data collection	✓	5
Participants	6	(*a*) Give the eligibility criteria, and the sources and methods of selection of participants	✓	6
Variables	7	Clearly define all outcomes, exposures, predictors, potential confounders, and effect modifiers. Give diagnostic criteria, if applicable	✓	6-7
Data sources/ measurement	8*	For each variable of interest, give sources of data and details of methods of assessment (measurement). Describe comparability of assessment methods if there is more than one group	✓	*8*
Bias	9	Describe any efforts to address potential sources of bias	✓	8
Study size	10	Explain how the study size was arrived at	✓	6
Quantitative variables	11	Explain how quantitative variables were handled in the analyses. If applicable, describe which groupings were chosen and why	✓	6-7
Statistical methods	12	(*a*) Describe all statistical methods, including those used to control for confounding	✓	8
		(*b*) Describe any methods used to examine subgroups and interactions		
		(*c*) Explain how missing data were addressed	Not Applicable	
		(*d*) If applicable, describe analytical methods taking account of sampling strategy	Not Applicable	
		(*e*) Describe any sensitivity analyses	Not Applicable	
**Results**				
Participants	13*	(a) Report numbers of individuals at each stage of study—eg numbers potentially eligible, examined for eligibility, confirmed eligible, included in the study, completing follow-up, and analysed	✓	9
		(b) Give reasons for non-participation at each stage	✓	9
		(c) Consider use of a flow diagram	Not Applicable	
Descriptive data	14*	(a) Give characteristics of study participants (eg	✓	9

For peer review only

For peer review only - http://bmjopen.bmj.com/site/about/guidelines.xhtml

		demographic, clinical, social) and information on exposures and potential confounders		
		(b) Indicate number of participants with missing data for each variable of interest	Not Applicable	
Outcome data	15*	Report numbers of outcome events or summary measures	✓	9-10
Main results	16	(*a*) Give unadjusted estimates and, if applicable, confounder-adjusted estimates and their precision (eg, 95% confidence interval). Make clear which confounders were adjusted for and why they were included	✓	10-12
		(*b*) Report category boundaries when continuous variables were categorized	Not Applicable	
		(*c*) If relevant, consider translating estimates of relative risk into absolute risk for a meaningful time period	Not Applicable	
Other analyses	17	Report other analyses done—eg analyses of subgroups and interactions, and sensitivity analyses	Not Applicable	
**Discussion**				
Key results	18	Summarise key results with reference to study objectives	✓	13
Limitations	19	Discuss limitations of the study, taking into account sources of potential bias or imprecision. Discuss both direction and magnitude of any potential bias	✓	15
Interpretation	20	Give a cautious overall interpretation of results considering objectives, limitations, multiplicity of analyses, results from similar studies, and other relevant evidence	✓	13-14
Generalisability	21	Discuss the generalisability (external validity) of the study results	✓	15
**Other information**				
Funding	22	Give the source of funding and the role of the funders for the present study and, if applicable, for the original study on which the present article is based	✓	22

*Give information separately for exposed and unexposed groups.

Note: An Explanation and Elaboration article discusses each checklist item and gives methodological background and published examples of transparent reporting. The STROBE checklist is best used in conjunction with this article (freely available on the Web sites of PLoS Medicine at http://www.plosmedicine.org/, Annals of Internal Medicine at http://www.annals.org/, and Epidemiology at http://www.epidem.com/). Information on the STROBE Initiative is available at www.strobe-statement.org.

For peer review only
